# Effects of Aroclor 1254 on Intestinal Immunity, Metabolism, and Microflora in Zebrafish

**DOI:** 10.3389/fnut.2022.929925

**Published:** 2022-07-13

**Authors:** Di-Hua Zhu, Fang-Hong Nie, Min Zhang, Wan Wei, Qing-Lang Song, Yao Hu, Dan-Ju Kang, Zhi-Bao Chen, Hong-Ying Lin, Jin-Jun Chen

**Affiliations:** ^1^Department of Veterinary Medicine, College of Coastal Agriculture, Guangdong Ocean University, Zhanjiang, China; ^2^College of Food Science and Technology, Guangdong Ocean University, Zhanjiang, China

**Keywords:** microbiome, PCBs (polychlorinated biphenyls), gut, metabolomics, zebrafish

## Abstract

Polychlorinated biphenyls (PCBs) are widely distributed environmental toxicants, whose biological toxicity is magnified step by step through the transmission of the food chain. However, there is little research about the effect of PCBs on intestinal epithelial barrier function. In this experiment, the effects of PCB exposure on the intestines of zebrafish were evaluated. Animals were exposed to Aroclor 1254 (5 μg/L, 10 μg/L, 15 μg/L). After 21 days, the changes in histology, enzyme biomarkers, intestinal microorganisms, and metabolomics were detected. The inflammation and oxidative stress in the intestines of zebrafish were observed. Additionally, there were significant changes in intestinal microbiota and tissue metabolism, most of which were associated with oxidative stress, inflammation, and lipid metabolism. The results showed that PCBs exposure resulted in intestinal inflammation and oxidative stress in zebrafish.Moreover, intestinal metabolites and intestinal microflora of zebrafish were also disturbed. This study verified that exposure can lead to intestinal damage and changes in intestinal metabolic capacity and microorganisms, enlightening the consequences of PCB exposure.

## Introduction

Polychlorinated biphenyls (PCBs) are one of the most representative persistent organic pollutants (POPs) (1). With excellent chemical stability and low electrical conductivity, PCBs are widely used as insulating media, plasticizers, lubricants, and flame retardants for transformers and capacitors (2). Later, it was verified to have teratogenic, carcinogenic, and mutagenic effects, as well as harmful effects on immune, reproductive, nervous, and endocrine systems (3, 4).

PCBs accumulate in the adipose tissue of living animals, resulting in biomagnification through the food chain (5). The most common way for humans to be exposed to these pollutants is to eat contaminated food. Consequently, the intestinal environment is a target organ worthy of further study. However, the effect of PCBs on intestinal epithelial barrier function was rarely investigated.

Zebrafish has been considered a model animal for the study of intestinal inflammation and the interaction between host metabolism and intestinal microflora owing to its small size, high reproductive ability, rapid development, and similar intestinal and immune functions to mammals (6, 7).

Increasing research has confirmed that intestinal microorganisms play a crucial role in regulating host immune homeostasis and metabolic function (8). Intestinal microflora diversity is a good indicator of host health, and low microbial community diversity can be repeatedly observed in patients with inflammatory bowel disease (9). The homeostasis of intestinal metabolism and the stability of intestinal microflora are critical for maintaining host health (10). Many studies have adopted the combination of metabolomics and macrogenomics to explore the correlation between metabolic changes and intestinal microflora changes, contributing to a new perspective for understanding the toxicity of environmental pollutants (11). However, there are few studies on the effects of PCB exposure on the intestine using metabolomics and macrogenomics.

This study aimed to evaluate the effects of PCB exposure on the intestines of zebrafish. Aroclor 1254 is one of the typical representatives of PCBs. Therefore, Aroclor 1254 of 5 μg/L, 10 μg/L, and 15 μg/L was selected in this experiment. Then, the PCB exposure experiment was conducted on adult zebrafish for 21 days to better reveal the chronic toxicity of PCBs to intestinal metabolism and microflora (bought for 5 months, adaptive feeding for half a month). The damage to zebrafish intestinal tissue caused by PCBs was assessed by biomarker monitoring and histological analysis. Metabolomics and macrogenomics maps were employed to determine the intestinal functional response to PCB exposure. Furthermore, the relationship between altered metabolites and intestinal bacteria was identified. These data provide new insights into the intestinal toxicity of PCBs.

## Materials and Methods

### The Raising of Zebrafish

Zebrafish (5 months old) was purchased from Shandong Xiyue Biotechnology Co., Ltd., with a uniform size, body length of 3 ± 0.5 cm, and average wet weight of 0.34 ± 0.05 g. Fish were temporarily raised in fully aerated water, which was equipped with a circulating water pump to ensure sufficient oxygen, accompanied by a water temperature of 25~28°C, light time of 14 h, and dark time of 10 h. The circulating water was filtered by reverse osmosis (pH 7.4~7.6) with an electrical conductivity of 500~550 μS/cm. Fish were adaptively fed every day for 2 weeks with special fish feed in a time-based manner. The residues at the bottom of the cylinder were removed on time to avoid the effect of the water quality.

### Experimental Grouping and PCB Exposure

The adapted zebrafish were randomly divided into 4 groups (Control group, H-PCBs group, M-PCBs group, L-PCBs group) and exposed for 21 days (50 fish in each glass tank, 10 L test solution). Each group has 300 fish. In the treatment group, PCBs were dispersed into the culture medium (Double distilled H_2_O) with a final concentration of 5 μg/L (L-PCBs), 10 μg/L (M-PCBs), and 15 μg/L (H-PCBs). The selection of these exposure concentrations was determined by the environmental-related concentrations (27~35 μg/kg) previously detected in mangrove sediments in our laboratory (12), as well as the previous literature on the toxicity of multi-PCBs to zebrafish (10 μg/L) in our laboratory (13).

The fish in the control group were cultured in PCBs-free water. During the experiment, the test solution in each tank was updated every 48 h (the test solution was prepared as mentioned above). Other conditions were consistent with the domestication period. All animal experiments were conducted following the guidelines for animal care and used in the laboratory of the National Institute of Health. All experiments involving animals were performed under the Guidelines for Care and Use of Laboratory Animals of Guangdong Ocean University and were approved by the Animal Ethics Committee of Guangdong Ocean University (Approval Number: 2019090504).

Three weeks after exposure, all zebrafish were sampled on the same day, washed with pure water (Meilunbio) 3 times, and immersed in ice water (<4°C) for euthanasia. Besides, different treatment methods were adopted according to different analysis methods. Simultaneously, the stool samples of the H-PCBs group, M-PCBs group, L-PCBs group, and control group were collected for macrogenomic analysis.

### Histological Observation Under the Influence of PCBs

The effects of PCB exposure on zebrafish intestinal tissue were detected using 24 fish (6 fish in each group). Specifically, 4% paraformaldehyde was fixed and embedded in paraffin. The section was 4 μm thick and stained with HE. Histological analysis was performed by optical microscope (SOPTOP, EX31).

### Evaluation of Biomarkers of Inflammation

In this part, 240 fish (10 for each repeat, 6 for each group) were selected for analysis. IL-8, IL-6, TNF-α, and IL-1 were adopted to evaluate the inflammation. RNA was extracted by TRIZOL, and cDNA was synthesized by reverse transcriptase R323-01 HiScript III RT Super Mix for qPCR (+gDNAwiper) (Vazyme, Beijing, China). Quantitative RT-PCR analysis was performed using SYBRGreen (Vazyme, China, Beijing). The amplification conditions of the reaction mixture were: pre-denaturation at 94°C for 30s, denaturation at 94°C, annealing at 55°C, annealing at 55°C, and then extension at 72°C for 10s, with a total of 40 cycles. The primers used are listed in Table 1. Furthermore, the 2-delta CT method was used to evaluate the results with β-actin as the reference gene, so as to determine the change in the relative expression of the gene.

**Table 1:** Primers.

**Gene**	**Sequence**	**Number**
TNF-α-F	CTGGATCTTCAAAGTCGGGTGTATGG	NM_212859.2
TNF-α-R	TTGTTGATTGCCCTGGGTCTTATGG	
IL-6-F	GTCTGCTACACTGGCTACACTCTTC	NC_007130.7
IL-6-R	CGTCCACATCCTGAACTTCGTCTC	
IL-1β-F	CCTGAACAGAATGAAGCACATCAAACC	NM_212844.2
IL-1β-R	GTAAGACGGCACTGAATCCACCAC	
β-actin-F	TGAATCCCAAAGCCAACAGAGAGAAG	NM_131031.1
β-actin-R	CCATCACCAGAGTCCATCACAATACC	
IL-8-F	AACATGGAGGTCATTGCCACTGTG	NM_001115068.1
IL-8-R	GGAGGTAGAATTTGGAGGGAGGGTAG	

### Biochemical Analysis

The enzyme-labeled biomarkers of functional reaction in intestinal tissue were determined by a commercial kit (Jiangsu enzyme-immunized MEIMIAN). The oxidative stress was evaluated using the activities of catalase (CAT), superoxide dismutase (SOD), glutathione (GSH), total antioxidant capacity (T-AOC), malondialdehyde (MDA), and reactive oxygen species (ROS). The intestinal permeability was obtained with the activities of diamine oxidase (DAO) and the D-lactic acid enzyme. Then, 200 fish (10 for each repeat, 5 for each group) were selected for measurement. The experimental operation was performed following the instructions of the kit (Rinse all intestinal contents with PBS).

### Metabonomic Analysis

A total of 240 fish (10 for each repeat, 6 for each group) were selected for metabonomic analysis. Metabolites were extracted by collecting the intestines of each repeated fish (14). Specifically, 50 mg of the solid sample or 100 μL of the liquid sample and 400 μL of extract (acetonitrile: methanol = 1:1) were added to a 1.5 mL centrifuge tube; after being vortex-mixed for 30 s, low-temperature ultrasonic extraction was performed for 30 min (5°C, 40 kHz); next, the sample was placed at −20°C for 30 min and centrifuged at 13,000 g, 4°C for 15 min to remove the supernatant; afterward, it was dried with nitrogen, 120 μL of the complex solution was re-dissolved (acetonitrile: water = 1:1), low-temperature ultrasonic extraction was performed for 5 min (5°C, 40 kHz), and centrifugation was conducted at 13,000 g, 4°C for 5 min. Finally, the supernatant was removed from the injection vial with intubation for analysis.

The instrument platform of this LC-MS analysis is ABSCIEX's ultra-high performance liquid chromatography-tandem time-of-flight mass spectrometry (UPLC-TripleTOF) system. Chromatographic conditions referred to the study of Zhao et al. (15). After the UPLC-TOF/MS analyses, the raw data were imported into the Progenesis QI 2.3 (Non-linear Dynamics, Waters, USA) for peak detection and alignment. The preprocessing results generated a data matrix composed of the retention time (RT), mass-to-charge ratio (m/z) values, and peak intensity. Metabolic features detected at least 80 % in any set of samples were retained. After filtering, minimum metabolite values were imputed for specific samples in which the metabolite levels fell below the lower limit of quantitation and each Metabolic feature was normalized by sum. Mass spectra of these metabolic features were identified using the accurate mass, MS/MS fragments spectra, and isotope ratio difference obtained in reliable biochemical databases such as the Human metabolome database (HMDB) (http://www.hmdb.ca/) and Metlin database (https://metlin.scripps.edu/). Concretely, the mass tolerance between the measured m/z values and the exact mass of the components of interest was ± 10 ppm. Concerning metabolites with MS/MS confirmation, only the ones with MS/MS fragment scores above 30 were considered confidently identified; otherwise, metabolites had only tentative assignments.

### Microbial Analysis

Regarding intestinal microflora analysis, 6 organisms were used for intestinal microflora analysis in each group. Total genomic DNA was extracted from fecal samples using FastDNA Soil Kit (MP Biomedicals, USA) in accordance with the manufacturer's agreement. The V3-V4 variable region of the 16s rRNA gene was amplified by PCR using 338 F and 806 R. The amplification procedure was detailed as follows. First, it was pre-denatured at 95°C for 3 min, with a total of 27 cycles (denatured 30s at 95°C, annealing at 55°C for 30s, extension at 72°C for 30s); then, it was stably extended at 72°C for 10mi, finally, it was preserved at 4°C (PCR instrument: ABI GeneAmp ®9700). The PCR reaction system was: 5×TransStart FastPfu buffer 4 μL, 2.5 mm dNTPs 2 μL, upstream primer (5 uM) 0.8 μL, downstream primer (5 uM) 0.8 μL, TransStart FastPfu DNA polymerase 0.4 μL, template DNA 10 ng, supplemented to 20 μL. There are 3 repeats in each sample.

The PCR products were detected and quantified by QuantiFluor fluorescence-ST blue fluorescence quantitative system (Promega). The samples were sent to Shanghai Meiji Biopharmaceutical Technology Co., Ltd for sequencing. The raw reads were deposited into the NCBI Sequence Read Archive (SRA) database (Accession Number: SUB11493756), and the associated BioProject number is PRJNA839159. The sequence data were divided into OTU, β diversity, principal component analysis (PCA), principal coordinate analysis (PCoA), and phylogenetic tree construction based on 97% similarity.

### Statistical Analysis

A multivariate statistical analysis was performed using ropls (Version1.6.2,http://bioconductor.org/packages/release/bioc/html/ropls.html) R package from Bioconductor on Majorbio Cloud Platform (https://cloud.majorbio.com). Principle component analysis (PCA) with an unsupervised method was completed to obtain an overview of the metabolic data and visualize general clustering, trends, or outliers. All of the metabolite variables were scaled to unit-variances before PCA. Orthogonal partial least squares discriminate analysis (OPLS-DA) was conducted to determine global metabolic changes between comparable groups. All of the metabolite variables were scaled to Pareto Scaling before OPLS-DA. The model validity was evaluated from model parameters R2 and Q2 to provide information for the interpretability and predictability, respectively, of the model and avoid the risk of over-fitting. Variable importance in the projection(VIP) was calculated in the OPLS-DA model. *p-*values were estimated with paired Student's *t*-test on Single dimensional statistical analysis.

Afterward, the metabolic pathways of differential metabolites were annotated by the KEGG database (https://www.kegg.jp/kegg/pathway.html), and the pathways involved in differential metabolites were obtained. Specifically, the Python software package scipy.stats was employed to analyze the pathway enrichment, and the biological pathway most related to the experimental treatment was determined by Fisher's accurate test.

## Results

### Effects of PCB Exposure on Intestinal Tissue

**Figure 1:**
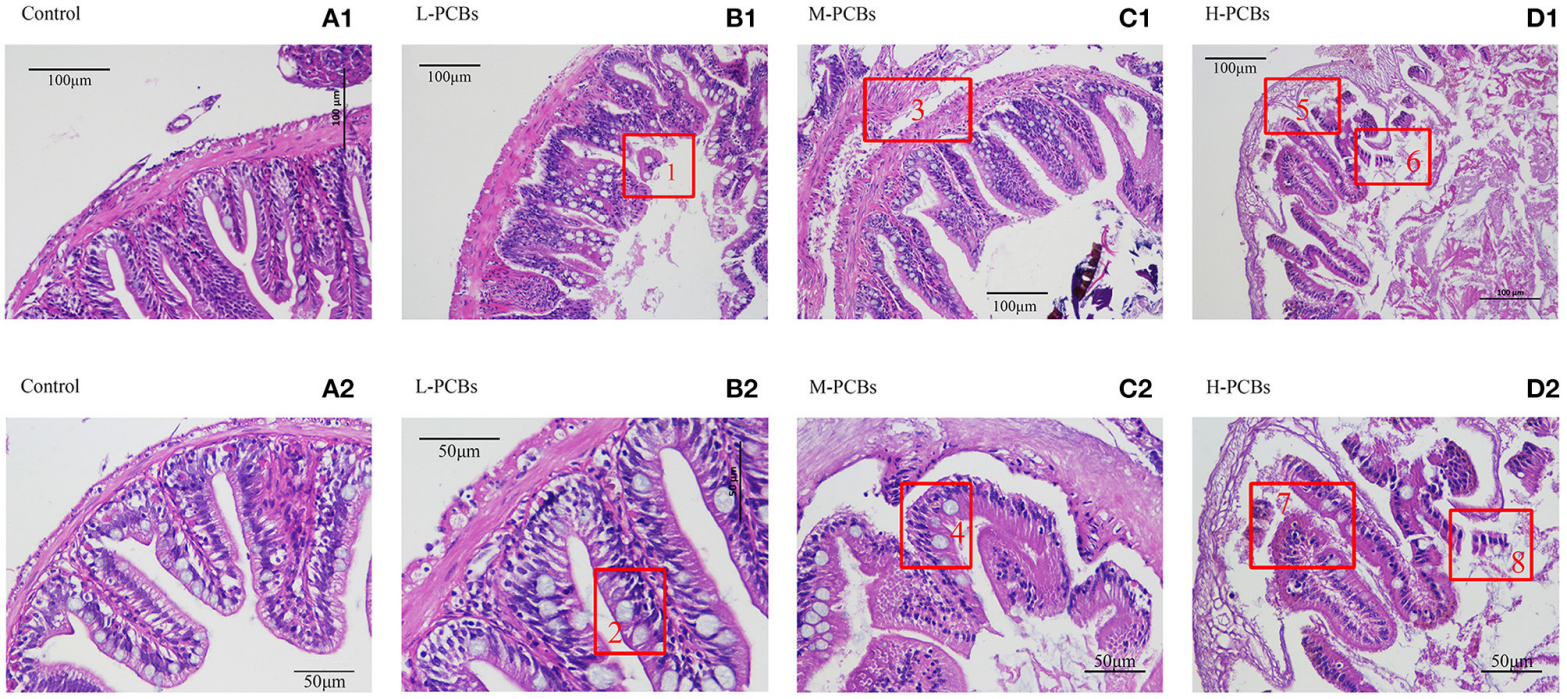
Intestinal tissue sections at different doses [**(A1,2)** Control, **(B1,2)** L-PCBs, **(C1,2)** M-PCBs, **(D1,2)** H-PCBs, 2.4.: Neutropenia, 1.6.8 intestinal villus injury, 3.7.5 epithelial injury, HE, 200X 400X].

Histological changes were observed in the PCBs-treated group 21 days after exposure. There was intestinal wall thinning in the L-PCBs group; intestinal villus injury in the M-PCBs group; villus injury, and epithelial injury in the H-PCBs group. The results demonstrated that the intestinal injury in the histological sections of each group was gradually aggravated and the intestinal wall gradually became transparent with the increase in the dose (Figure 1).

### Increased Intestinal Oxidative Stress and Permeability Induced by PCB Exposure

**Figure 2:**
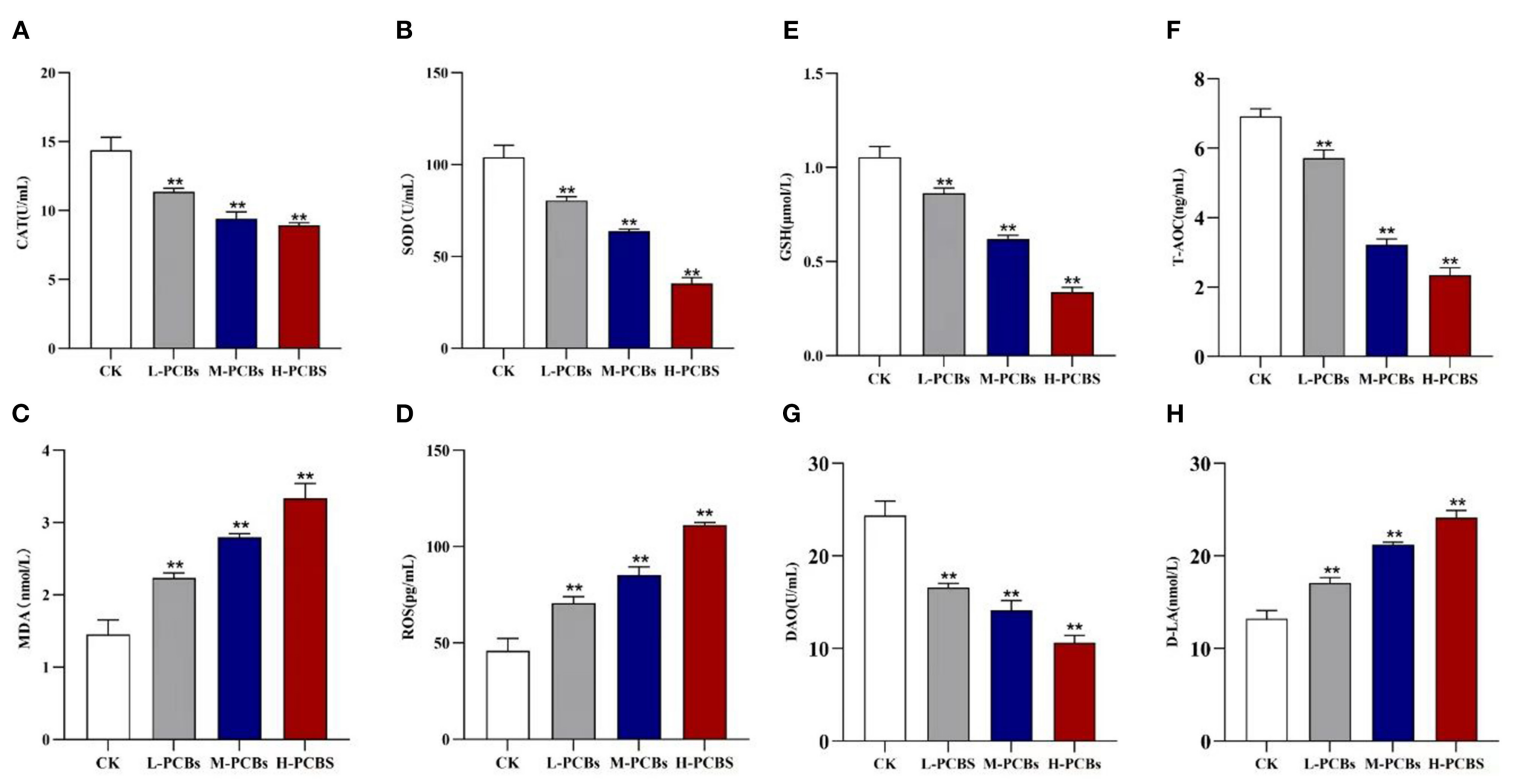
Change map [**(A)**. CAT **(B)**.SOD **(C)**. MDA **(D)**. ROS **(E)**.GSH **(F)**. T-AOC **(G)**. DAO **(H)**. D-LA, *P < 0.05, **P < 0.01 compared to control group].

The activities of CAT and SOD in the dissected intestinal tissue were measured to investigate the intestinal oxidative stress caused by PCB exposure (Figures 2A,B). Compared with the control group, the CAT activity in L-PCBs, M-PCBs, and H-PCBs groups significantly decreased by 20.9, 34.4, and 38.0%, respectively (*P* < 0.01); the SOD activity in L-PCBs, M-PCBs, and H-PCBs groups dramatically decreased by 22.7, 38.9, and 65.9%, respectively (*P* < 0.01); the content of MDA in L-PCBs, M-PCBs, and H-PCBs groups remarkably increased by 53.7, 92.2, and 129.2%, respectively (*P* < 0.01); the content of ROS in L-PCBs, M-PCBs, and H-PCBs groups considerably increased by 53.9, 85.9, and 142.2%, respectively (*P* < 0.01); the GSH activity in L-PCBs, M-PCBs, and H-PCBs groups substantially decreased by 18.1, 41.4, and 68.0%, respectively (*P* < 0.01); the T-AOC ability of L-PCBs, M-PCBs, and H-PCBs groups noticeably decreased by 17.4, 53.5, and 66.2%, respectively (*P* < 0.01). Besides, the levels of DAO and D-lactate in the zebrafish intestine were measured to evaluate the effect of PCB exposure on intestinal permeability (Figures 2C,D). Compared with the control group, the DAO in L-PCBs, M-PCBs, and H-PCBs groups significantly decreased by 31.9, 42.1, and 56.4%, respectively (*P* < 0.01); D-lactic acid in L-PCBs, M-PCBs, and H-PCBs groups surprisingly increased by 28.7, 60.4, and 82.2%, respectively (*P* < 0.01).

### Relative Expression of Target Gene MRNA

**Figure 3:**
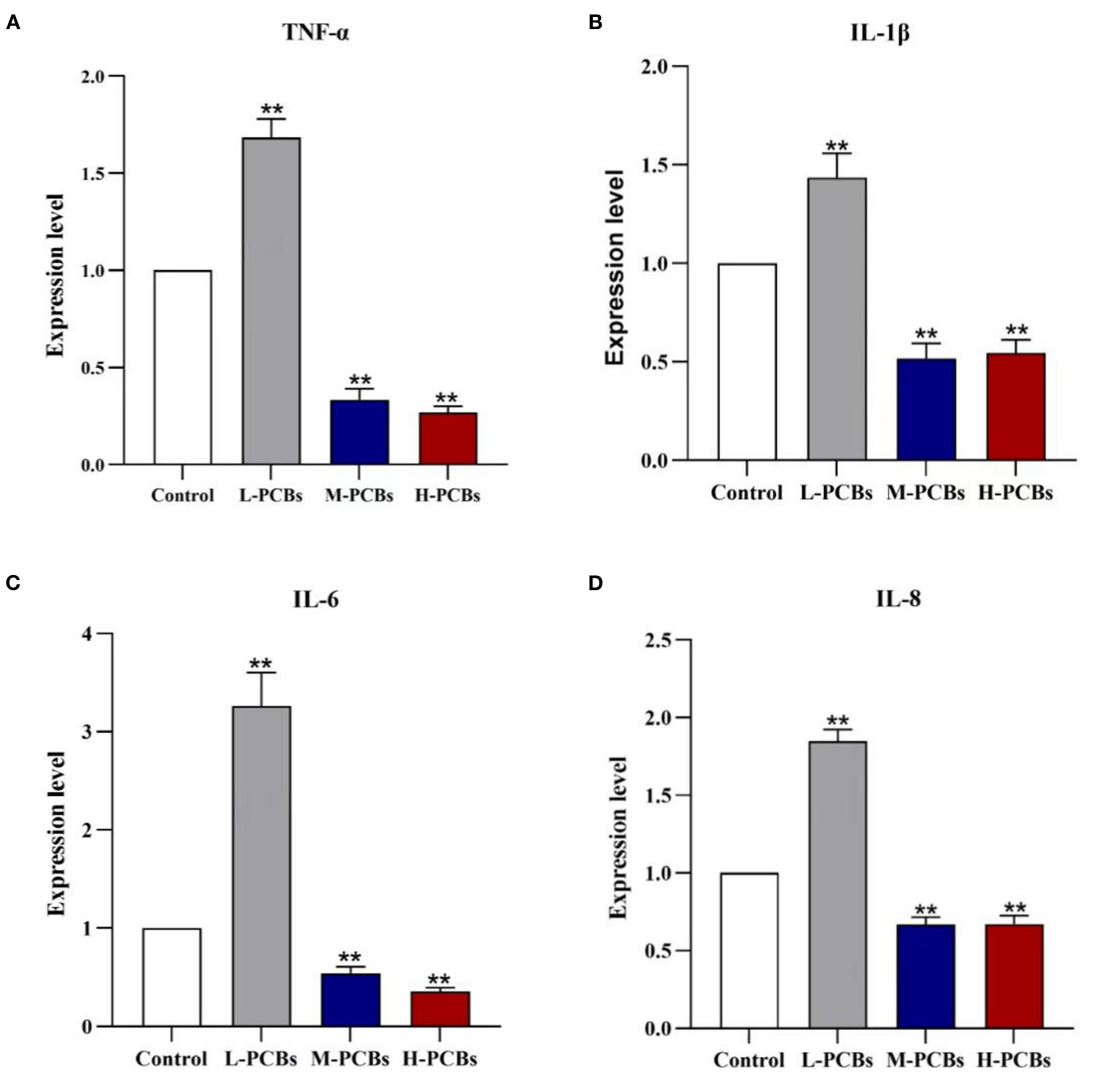
Changes of cytokines (TNF-α?IL-1β?IL-6 and IL-8) mRNA expression in zebrafish exposure to [**(A)**. TNF-α **(B)**. IL-1β **(C)**. IL-6 **(D)**.IL-8, *P < 0.05, **P < 0.01 compared to control group].

There has been some evidence that PCB126 triggers inflammation in intestinal tissue (9). Therefore, the gene expression of immune-related markers in the intestinal tract of zebrafish was detected using qPCR. In summary, Aroclor 1254 elicited a similar response in the intestine (Figure 3). In the low-dose group, Aroclor 1254 significantly boosted the expression of IL-8, IL-6, TNF- α, and IL-1, respectively (1.85 times, *P* < 0.01). Additionally, Aroclor 1254 reduced the expression of IL-8, IL-6, TNF- α, and IL-1 factors in middle and high dose groups.

### Metabolic Group Changes Induced by PCB Exposure

**Figure 4:**
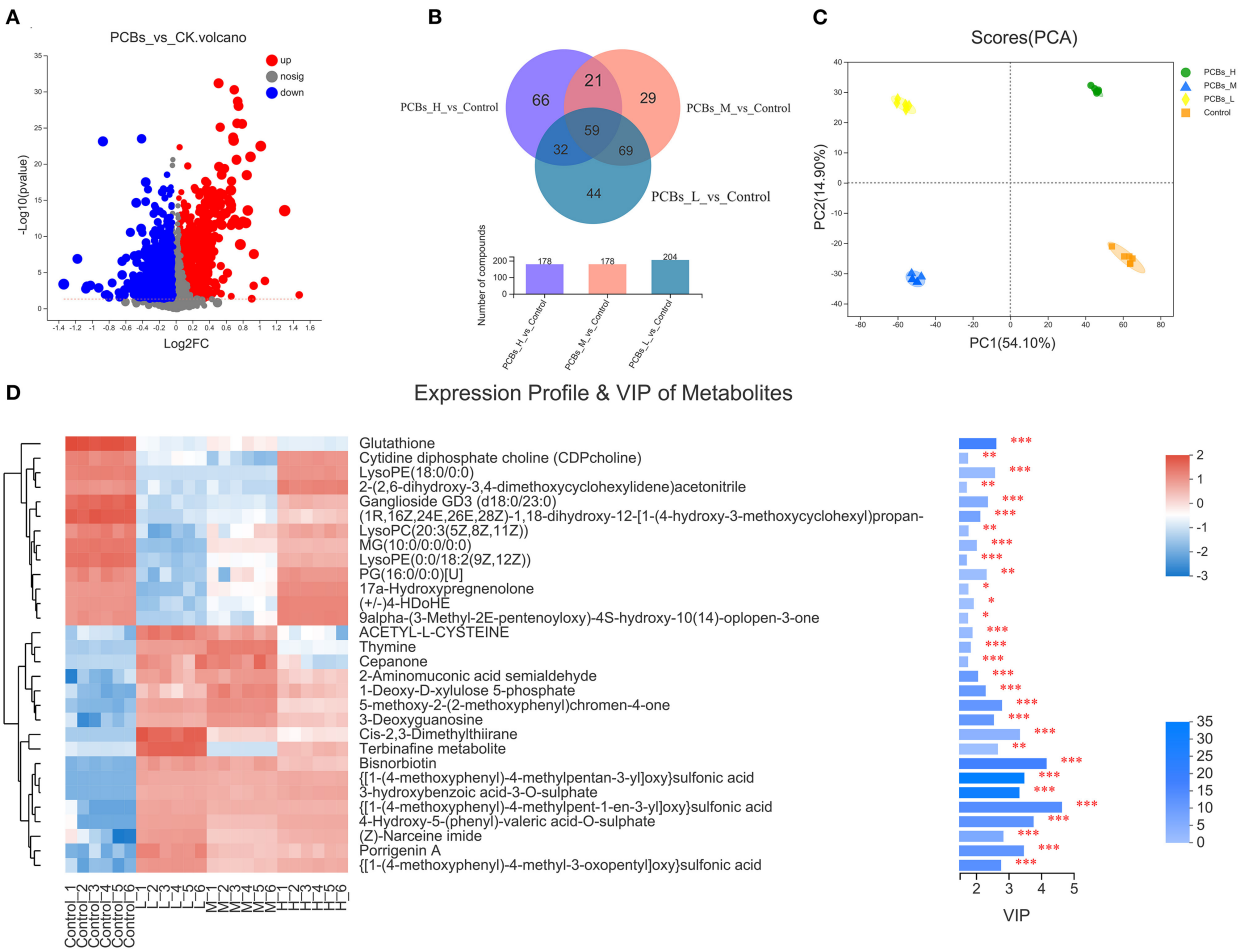
Change map of the intestinal metabolic group [**(A)**. Metabolite changes **(B)**. Venn diagram of metabolites in each experimental group **(C)**. PLS-DA diagram of metabolites in each experimental group **(D)**. Hierarchical clustering heat map, *P < 0.05, **P < 0.01, ***P < 0.001].

The changes in the intestinal metabolic group after PCB exposure were explored by the metabonomic analysis. The results revealed significant changes in 132 metabolites due to PCB exposure (*P* < 0.05, VIP>1) (55 metabolites increased and 77 metabolites decreased) (Figure 4A). A few metabolites (29.8%) were shared among H-PCBs, M-PCBs, and L-PCBs treatment groups (Figure 4B).

The PLS-DA diagram exhibited a clear separation of metabolic spectra between the control group and each PCBs-treated group (Figure 4C). Additionally, the coefficients and VIP values of OPLS-DA were calculated to further determine the contribution of metabolites to metabonomic changes caused by PCB exposure. Figure 4D illustrated that 30 metabolites had a significant positive contribution to the change of metabolite (VIP>1). The hierarchical clustering heat map presented a similar pattern of metabolite changes in each group (Figure 4D), supporting the PLS-DA score map.

### Disturbance of Intestinal Microbiota Caused by PCB Exposure

**Figure 5:**
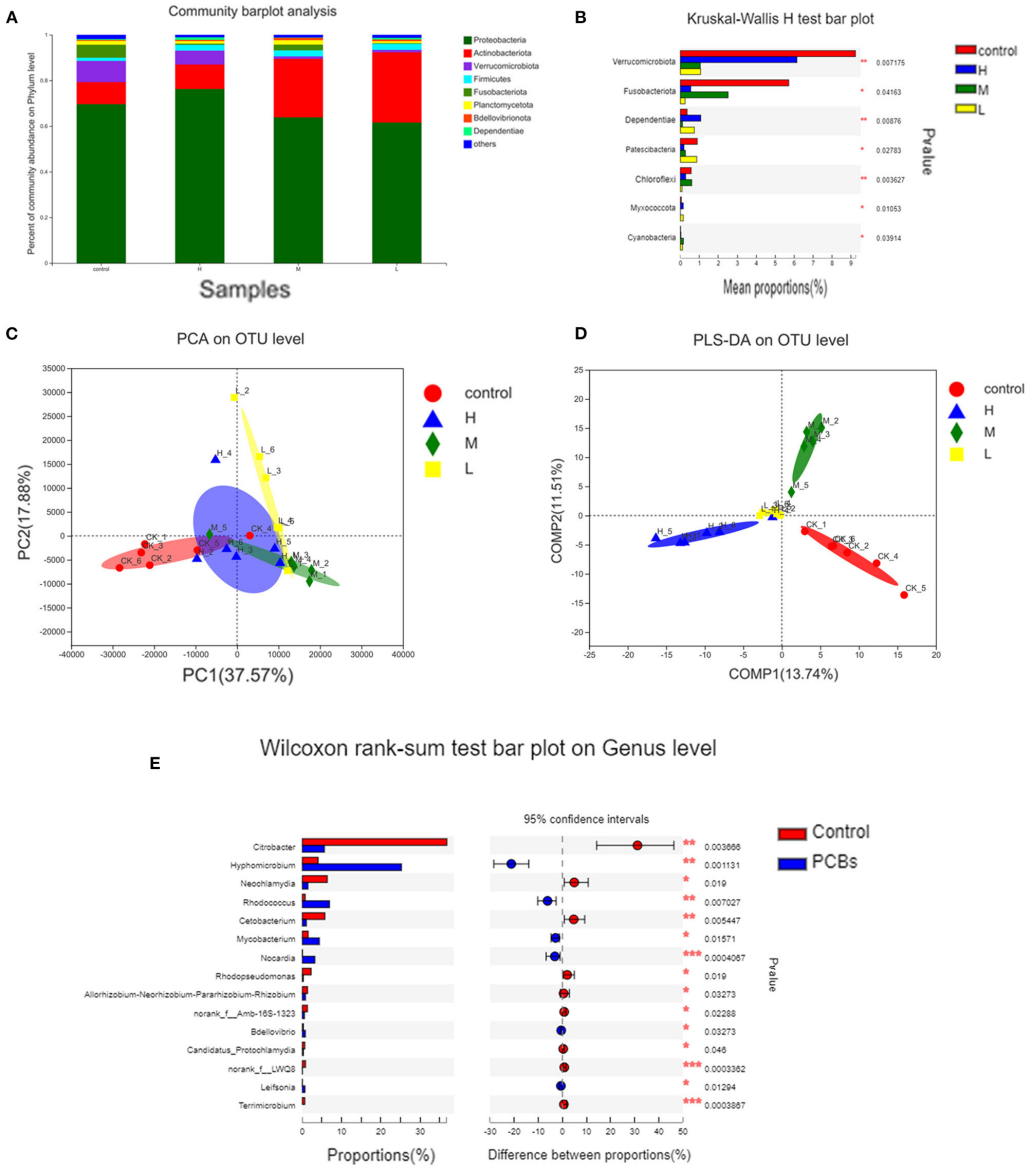
Analysis of intestinal microorganisms of zebrafish treated with different doses of PCBs [**(A)**. The microbial composition of each dose group **(B)**. The difference in composition of each dose group was analyzed by **(C)**. PCA analysis **(D)**. PLS-DA analysis **(E)**. The difference in genus level between the control group and the experimental group].

The changes in intestinal microflora of zebrafish exposed to PCBs were detected through the macrogenomic analysis of high-throughput sequencing. The intestinal microflora of PCBs-treated zebrafish and control zebrafish were mainly *Proteobacteria* and *Actinobacteriota* (Figure 5A). Compared with the control group, the number of *Verrucomicrobiota* and *Fusobacteriota* significantly decreased (*P* < 0.05). The number of *Dependentiae* remarkably increased in the H-PCBs group (*P* < 0.05). *Cyanobacteria* increased in the M-PCBs group. *Myxococcota* increased in the L-PCBs group, and there was a similar trend in the H-PCBs group (Figure 5B).

The PCA diagram demonstrated that PCB exposure at the genus level induced three unique intestinal microflorae depending on the dose (Figure 5C). The control group and PCBs-treated group were successfully separated, and the two principal components (PC1 and PC2) explained 37.57% and 17.88% of the variation, respectively. The PLS-DA analysis also confirmed the significant isolation of intestinal flora at the genus level between the control group and different dose PCBs treatment groups (Figure 5D).

The differences between groups were analyzed to demonstrate significant changes in 15 genera. Then, the effect of PCBs on the intestinal flora was further determined. Compared to the control group, 11 bacterial genera were significantly higher, while 4 bacterial genera were significantly down-regulated. Most of the 15 genera belonged to Fusobacteria and *Verrucomicrobiota* (Figure 5E).

### The Relationship Between Altered Metabolites and Microorganisms

**Figure 6:**
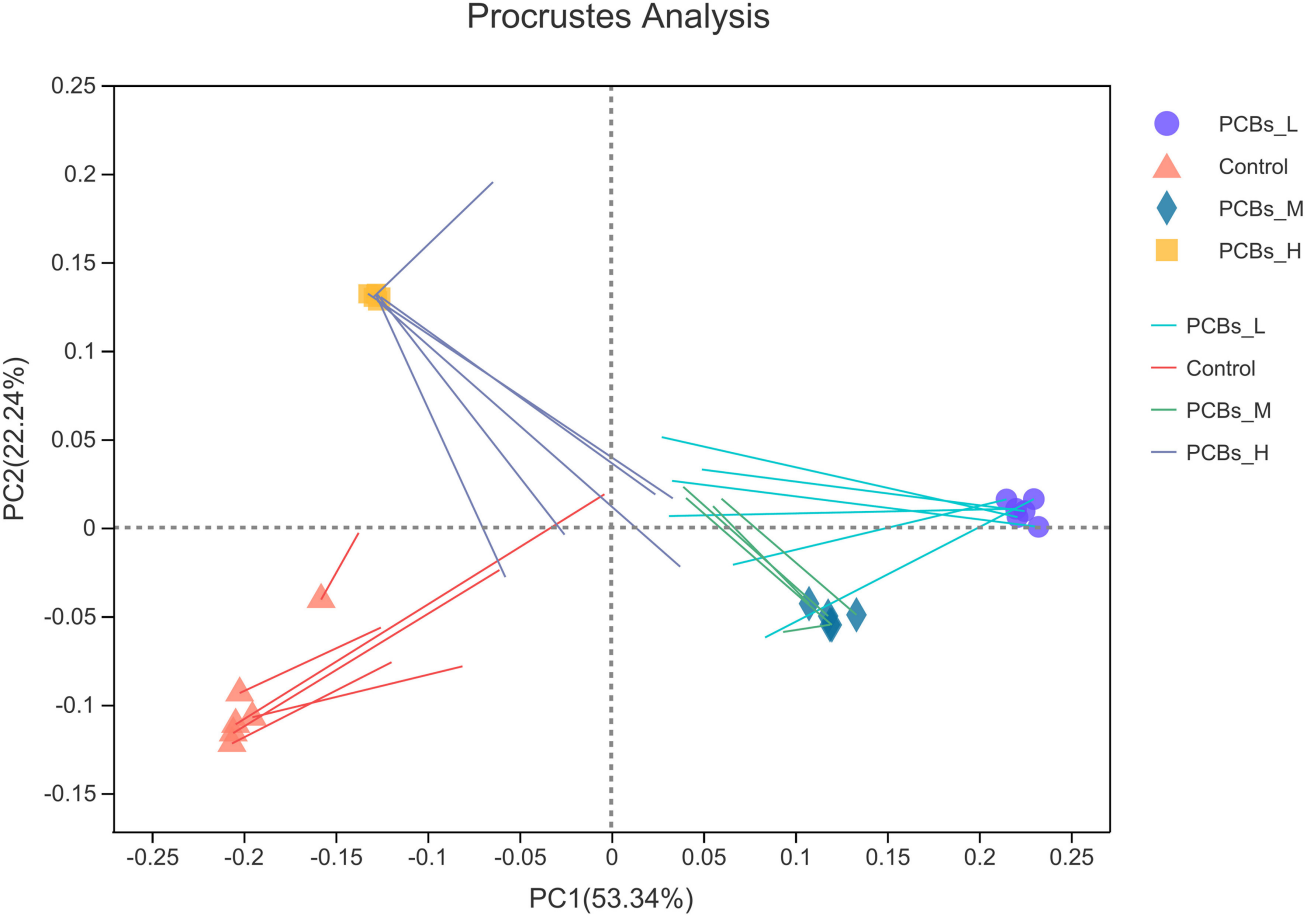
Platts analysis.

PCB exposure induced significant changes and classification disorders in the intestinal microbial metabolic group. Thus, the potential relationship between metabolites and bacteria was deeply investigated in this study. The results revealed that the characteristics of intestinal microorganisms and the expression of metabolites were consistent among different groups (*P* < 0.01) (Figure 6).

## Discussions

Gut microbes play a regulatory role in the digestive system and host immunity. Gut microbes in fish are increasingly studied as the organism's “second genome”. A major source of exposure to PCBs is the consumption of contaminated food.

The accumulation of PCBs in intestinal tissue may cause various adverse reactions. The present study demonstrated that exposure to PCBs in zebrafish led to intestinal oxidative stress, intestinal inflammation in the low-dose group, and immunosuppression in the medium-high-dose group. Histological observations suggested that the PCBs-treated zebrafish exhibited an inflammatory response in the intestinal tissue, accompanied by thinning of the intestinal wall, damage to the villi, and epithelial damage, as well as reduction of goblet cells and shallow crypts.

Furthermore, the reduction in glutathione in the gut metabolome was consistent with a reduction in GSH enzyme activity in antioxidant parameters. These two findings confirmed that the activity of CAT, SOD, GSH enzymes, and total antioxidant capacity (T-AOC) in the gut of fish treated with PCBs were significantly weakened. Elevated levels of malondialdehyde (MDA) and reactive oxygen species (ROS) implied that oxidative stress was caused by PCBs. Glutathione is an essential intracellular antioxidant that prevents the uncontrolled formation of free radicals and reactive oxygen species (ROS) and inhibits their response to DNA, proteins, and lipids. The change of the MDA content in the body can reflect the degree of lipid peroxidation in the body and the degree of cell damage. As a crucial regulatory metabolic agent and antioxidant in cells, GSH can scavenge oxygen-free radicals, enhance the activity of antioxidant enzymes, and improve the body's antioxidant defense capacity. ROS has a high degree of activity and oxidative ability, acting on unsaturated fatty acids and increasing the content of MDA (the final product of lipid peroxidation). If these substances cannot be removed in time, they will damage the body at the molecular, cellular, and tissue organ levels. SOD and CAT are the main antioxidant enzymes of the body. They can remove excess ROS in the body and maintain the body's oxidative balance. The level of its activity indicates the strength of the body's antioxidant capacity. This is closely correlated with kidney disease, blood system disease, cardiovascular disease, and endocrine system disease. PCBs can induce elevated intracellular ROS levels (16) and lead to inflammation and oxidative stress (17).

Various toxic effects of PCB exposure in mammals are related to the oxidative stress injury in the body, involving the decrease in the body's main antioxidant enzyme activities, the increase in the lipid peroxidation product malondialdehyde, and the increase in the body's reactive oxygen species. After POPs enter the biological system, they will undergo a series of oxidation processes and may synthesize some active molecules including reactive ROS. Small amounts of ROS are required to enhance internal defenses against pathogens. However, a series of damage occurs when the production of reactive oxygen species is excessive. In this work, Aroclor 1254 was discovered to increase O^−2^ production compared to controls, consistent with the results of Coteur et al. (18). Wang Junling et al. reported that two carcinogenic polycyclic aromatic hydrocarbons induced the decrease in SOD activity and the increase in MDA content in the liver of Bufo bufo gargarizans, owing to the pathological changes in the body (19). Lin P H et al. revealed that a certain dose of TCDD significantly drove excessive ROS production in rat ovarian granulosa cells, boosted the content of lipid peroxidation product MDA, and lowered the activity of GSH, suggesting that the apoptosis of ovarian granulosa cells induced by TCDD may play a role through oxidative damage (20). PCBs reduced the antioxidant capacity and increased the lipid peroxidation products of zebrafish, resulting in the imbalance of the antioxidant system and serious oxidative damage.

The increase in long-term inflammation and oxidative stress in the intestinal tract is closely related to the imbalance of intestinal microflora and metabolic disorders (21, 22). The imbalance of intestinal flora can provoke immune dysfunction and the destruction of the intestinal barrier, leading to disease (23). After being treated with different concentrations of PCBs for 21 days, the intestinal flora significantly changed both at the gate level and the genus level. In this study, the intestinal microflora of zebrafish was dominated by *Proteobacteria* and *Actinobacteriota* in the PCBs-treated group and control group, consistent with the previous study of zebrafish (8). However, their relative abundance remarkably changed after exposure to PCBs. Compared with the control group, *Fusobacteriota* and *Verrucomicrobiot*a dramatically decreased. *Fusobacteriota* metabolizes carbohydrates (including mucin) into short-chain fat butyrates and thus brings many benefits to the host, involving providing energy to gastrointestinal cells, acting as anticancer and anti-inflammatory agents, and inhibiting potential freshwater fish pathogens. The decrease in the proportion of *Fusobacteriota* has a noticeably negative effect on the intestinal energy supply of the host and the maintenance of normal immune system homeostasis (24).

The number of TM6 (*Dependentiae*) and M-PCBs *Chloroflexi* significantly increased in the H-PCBs group. These flora disorders further verify the inflammation caused by PCB exposure. Intestinal inflammation may also trigger changes in intestinal permeability (25). Accordingly, the intestinal wall became thinner, and intestinal permeability increased in zebrafish treated with PCBs on the basis of reducing DAO and increasing D-lactic acid.

PCBs-treated zebrafish exhibited intestinal metabolic disorders, including amino acid metabolism and fat metabolism. For example, the metabolites related to nine amino acids (such as proline, leucine, lysine, threonine, alanine, phenylalanine, glutamine, tyrosine, and ornithine) and six lipid metabolites (such as propylene glycol, linoleic acid, palmitic acid, carnitine, triglyceride, and TMAO) in the intestines of zebrafish treated with PCBs changed significantly.

Similarly, disorders of amino acid metabolism and fat metabolism were observed in zebrafish treated with PCB126 (9). Mice exposed to PCBs also demonstrated disturbance of lipid metabolism (26, 27). Additionally, some of these altered metabolites were highly associated with intestinal microorganisms.

## Conclusions

This study sheds light on investigating the effects of PCB exposure on the zebrafish gut. After exposure to PCBs, significant histological changes and enzyme marker changes associated with intestinal inflammation and oxidative stress were observed in the zebrafish gut. Meanwhile, significant changes in the gut microbiota and gut metabolic profile were observed as a result of PCB exposure, most of which were correlated with gut inflammation and lipid metabolism. Furthermore, zebrafish have been widely used as a model to research intestinal diseases. Therefore, the findings of this study highlight the potential health risks of PCBs exposed to the gut and warrant further investigation. Our results lay a foundation for the research of the mechanisms by which PCBs affect the gut system.

## Data Availability Statement

The datasets presented in this study can be found in online repositories. The names of the repository/repositories and accession number(s) can be found below: https://www.ncbi.nlm.nih.gov; PRJNA839159.

## Ethics Statement

All experiments were approved by the Ethics Committee of Animal experiment of Guangdong Ocean University.

## Author Contributions

D-HZ: conceptualization, methodology, investigation, data curation, supervision, and writing-original draft. F-HN: conceptualization, methodology, investigation, supervision, and writing—review and editing. MZ, WW, Q-LS, and YH: investigation. D-JK: methodology. Z-BC: methodology, supervision, and writing-review and editing. H-YL: methodology and supervision. J-JC: conceptualization, methodology, data curation, supervision, funding acquisition, and writing—review and editing. All authors: discussed, reviewed, and approved the final report.

## Funding

Marine Economic Development Project of Guangdong Province Special Fund for Promoting High-quality Economic Development (Contract Grant Number: GDOE2019A52). Guangdong Postgraduate Education Innovation Project (2020SFJD001), and International Science and Technology Cooperation Project of Guangdong Province (Contract Grant Number: 2016A050502062).

## Conflict of Interest

The authors declare that the research was conducted in the absence of any commercial or financial relationships that could be construed as a potential conflict of interest.

## Publisher's Note

All claims expressed in this article are solely those of the authors and do not necessarily represent those of their affiliated organizations, or those of the publisher, the editors and the reviewers. Any product that may be evaluated in this article, or claim that may be made by its manufacturer, is not guaranteed or endorsed by the publisher.
